# The moderating effect of alexithymia on the relationship between stress and cyberchondria

**DOI:** 10.3389/fpsyt.2022.1043521

**Published:** 2022-10-25

**Authors:** Yi Zhou, Lisha Dai, Yunlong Deng, Hongling Zeng, Lifeng Yang

**Affiliations:** ^1^Department of Child-Adolescent Psychology, Wuhan Mental Health Center, Wuhan, China; ^2^Department of Child-Adolescent Psychology, Wuhan Hospital for Psychotherapy, Wuhan, China; ^3^Department of Psychosomatic, Wuhan Mental Health Center, Wuhan, China; ^4^The Third Xiangya Hospital, Psychosomatic Health Institute, Central South University, Changsha, China

**Keywords:** cyberchondria, alexithymia, stress, health-related online search, emotions

## Abstract

**Objectives:**

The pandemic has increased the level of perceived stress and provided a fertile soil for Cyberchondria. This study aims to investigate the relationship between stress, cyberchondria, and alexithymia.

**Methods:**

This cross-sectional study used convenience sampling to carried out on a total 1,117 college students (female/male:536/581; mean age: 19.95 ± 1.32). Cyberchondria Severity Scale (CSS), Toronto Alexithymia Scale (TAS) and Short Depression Anxiety Stress Scales (DASS-21) were used to assessed the present study variables.

**Results:**

Significant differences emerged for CSS scores by gender (*t* = 3.74, *p* < 0.01) and had any comorbid disease (*t* = 2.47, *p* < 0.05), The Pearson correlation showed Cyberchondria has a significant positive correlation with stress and alexithymia (*r* = 0.50, *p* < 0.01, *r* = 0.36, *p* < 0.01). Furthermore, the regression analysis described that stress (β = 0.50, *p* < 0.01) and alexithymia (β = 0.36, *p* < 0.01) were the significant positive predictors of cyberchondria. Moreover, moderation analysis showed that alexithymia significantly strengthened the association between stress and cyberchondria (*F* = 107.20, *p* < 0.01).

**Conclusion:**

The study will help medical practitioners to understand how stress and alexithymia can cause an increase in cyberchondria. This will help them to elaborate operational indications for prevention and psychological support.

## Introduction

The long incubation period and strong infectivity of coronavirus have become a major public health emergency worldwide (1), public emergencies may affect the health, safety and wellbeing of individuals (2). Due to the suddenness of coronavirus, people have little understanding of it and insufficient coping ability (3). As a health-related stress event, the COVID-19 pandemic brings health-related anxiety, which increases the pressure borne by people. Restrictive policies adopted by countries to slow the spread of infection have significantly affected people’s education, work, and social interactions (4), which further increasing the public physical and psychological stress (5, 6).

During repeated lockdowns and quarantines, the Internet became the primary source of health information. In the sample of Italian during the COVID-19 pandemic, nearly one-third of people, increased the habit of health-related online searches (7). However, a survey reported that, more than 75% of international web-users search health-related information online (8), 71.1% of individuals who searching health related information online become more worried (9). During the COVID-19 pandemic, cyberchondria was more prominent among people who used social media as the primary source of information about COVID-19.

The term Cyberchondria (10) is derived from “cyber” and “hypochondriasis,” where “cyber” refers to Internet use and “hypochondriasis” refers to pathological health anxiety. Cyberchondria refers to excessive, maladaptive, and repetitive online searches related to health, which lead to the impact of daily life and cause distress (8). Cyberchondria can be defined as a continuous dimensional structure, ranging from mild to severe psychopathological behavior patterns. Severe cyberchondria will cause psychological distress, social malfunction, overwhelming strain medical resources, and deteriorated doctor-patient relationship (3).

Many studies (3, 8, 11) have confirmed the association between health anxiety and cyberchondria. A systematic review and meta-analysis found strong relationship between health anxiety and cyberchondria (*r* = 0.62) (12). In the majority of individuals, health anxiety seems to be the main phenomenon leading to online health information search to alleviate this underlying health anxiety, but it is usually ineffective and makes it worse. Besides problematic Internet use (PIU), obsessive-compulsive disorder (OCD) and intolerance of uncertainty also have important relationships with cyberchondria. A recent review has integrated a cognitive-behavioral model of cyberchondria suggested that maladaptive personality features, health anxiety, intolerance of uncertainty and faulty meta-cognitions are predisposing factors for cyberchondria (12).

According to literature data, the researchers found that several factors, including personality traits, resilience, coping strategies, and alexithymia (13), played an important role in the impact of stressors on an individual’s mental health during the coronavirus pandemic (14). Alexithymia can be considered as a vulnerability factor to stress (15). Alexithymia is an impaired ability to identify, describe and differentiate one’s feelings, and a cognitive style that is utilitarian and externally oriented (16). Alexithymia was first seen in patients with psychosomatic symptoms, and a large proportion of patients experience somatic symptom but are unable to clearly express their feelings to the clinicians (17). Individuals with alexithymia have difficulty regulating emotional dysregulation and managing stress (18). The limited differentiation of emotional states and the existence of decoupling of implicit and explicit emotional responses (19) in alexithymia seem to actually cause patients great difficulty with regulating and resolving negative affect. As a result, the prevalence of affective psychiatric disorders such as major depressive disorder, generalized anxiety disorder, and posttraumatic stress disorder has increased in this population. Furthermore, difficulties with emotion regulation associated with alexithymia appear to be associated with an increase in addictive behaviors, such as substance addiction (20), excessive mobile phone and internet use (21).

Individuals with alexithymia are difficult to distinguish their own body sensory and emotional experience, leading to excessive attention to body feeling (22). Moreover, they are more difficult to regulate their emotions, especially when dealing with the pressure, which can cause physiological arousal and emotional feelings mismatch (16). As a result, individuals are more likely to be troubled by their own physical reactions and emotional experiences, which increases health-related online searches and leads to the symptoms of cyberchondria.

As state here, there are many studies found that Cyberchondria has relationships with health anxiety, PIU, and symptoms of OCD (3). But, there has few study explores the relationship between stress, cyberchondria, and alexithymia specifically. As mentioned above in review, we formulate the following research hypotheses:

(1): Stress will be the significant positive predictor of cyberchondria;

(2): Alexithymia will be the significant positive predictor of cyberchondria.

(3): Alexithymia will play a moderating role in the relationship between stress and cyberchondria such that an increase in Alexithymia will make this relationship stronger.

## Materials and methods

### Participants and procedure

The present study employed a cross-sectional study design. The participants were recruited through convenience sampling from college students in China. Two university students in Wuhan and Changsha were considered for collection, taking into consideration the convenience of the study team. All these university students were fluent in their ability to read, write, and comprehend Chinese and gave written informed consent for the study were included in the study. All tests were performed by two trained masters in psychology. This study received ethical approval from the hospital ethical committee.

After careful data screening, questionnaires with missing items or random responses were removed. Finally, we had 1,117 valid responses (92.93%), the sample included 536 females (48%) and 581 males (52%) with a mean age of 19.95 (±1.32). Moreover, data was collected from undergraduates (25.9% freshman, 33% sophomore, 37.2% junior, 3.9% senior). In addition to this, about 58.2% of participants belonged to urban areas, while 41.8% belonged to rural areas.

Power analysis for the sampling adequacy using *A Priori* method with α = 0.05 and a small effect size (0.2) also confirmed that the sample size of 1,000 is sufficient to draw inferences from data in this study.

### Measures

Three self-report scales were administered:

1. *The cyberchondria severity scale (CSS)* used in the research was developed by McElroy and Shevlin (23). The instrument uses 33 items to measure the severity of cyberchondria. It consists of 5 subscales (compulsion, distress, excessiveness, reassurance, and lack of trust in medical professionals). Each item is rated on five-point scale ranging from 1 (never) to 5 (always). A higher score indicates a greater severity of cyberchondria. In this study, the internal consistency (Cronbach’s α) of the CSS was 0.95.
2. *The Toronto alexithymia scale (TAS-20)* used in the research was developed by Bagby et al. (24). The instrument uses 20 items to measure the alexithymia. It consists of 3 subscales: difficulty identifying feelings (DIF); difficulty describing felling (DDF); and externally oriented thinking (EOT). Each item is rated on five-point scale ranging from 1 (do not agree) to 5 (agree very much). In this study, the internal consistency (Cronbach’s α) of the TAS-20 was 0.89.
3. *The shortform version of Depression Anxiety Stress Scales (DASS-21)* used in the research was developed by Lovibond et al. (25). The DASS-21 consists of 3 subscales: anxiety, depression, and stress. Each item is rated on 4-point Likert scale, ranging from 0 (Never) to 3 (Almost Always). In order to assess stress, the 7-item subscales were used. In this study, the internal consistency (Cronbach’s α) of the Stress subscales was 0.89.

### Data analysis

SPSS 26.0 version was used to analyze the data. All demographic and clinical variables were examined for deviations from the Gaussian distribution using the Kolmogorov-Smirnov test. The Harman’s single-factor test was used to evaluate common method variance. Descriptive statistics were performed on the demographic variables and psychometric outcomes, and Pearson correlation was used to explore the relationship between variables. Haye’s Process tool in SPSS (using model 1) was used for mediation analysis to test the moderating effect of the alexithymia on the relation between Stress and Cyberchondria. Internal consistency of the scales was calculated using Cronbach’s alpha.

## Results

According to the Harman’s single-factor test: After unrotated exploratory analysis of all measurement items, there were 9 common factors with characteristic root greater than 1, and the first common factor explained 31.29% of the variation. No single factor could explain most of the variation, and there was no common method bias in this study.

The study sample of *n* = 1,117 comprised undergraduate, university students of which 536 (48%) were female, and 581 (52%) were male participants. Table 1 reports on the demographic characteristics and associated scores on the CSS. According to the results, the independent sample *t*-test of CSS showed that there was statistically significant difference in gender (*t* = 3.74, *p* < 0.01). The mean of CSS was higher in males than females. Additionally, participants that reported had any comorbid disease (*t* = 2.47, *p* = 0.015) showed significant higher CSS scores. No differences emerged for CSS scores by grade (*F* = 0.17, *p* = 0.917), age (*F* = 0.50, *p* = 0.874), and hometown (*t* = −1.72, *p* = 0.086).

**TABLE 1:** Demographic variables showing a statistically significant difference on the CSS.

Variables		Mean (±SD) – *N* (%)	CSS Mean (± SD)	*P*-value
Total sample		1,117	72.50 ± 21.47	−
Age^a^		19.95 (±1.32)	−	0.874
Gender^b^				**<0.01**
	**Male**	**581 (52.0%)**	**74.80 ± 21.78**	
	**Female**	**536 (48.0%)**	**70.01 ± 20.87**	
Native place^b^				0.086
	Urban areas	650 (58.2%)	71.56 ± 21.42	
	Rural areas	467 (41.8%)	73.80 ± 21.50	
Grade^a^				0.917
	Freshman	289 (25.9%)	72.76 ± 22.04	
	Sophomore	369 (33.0%)	72.01 ± 20.74	
	Junior	415 (37.2%)	72.58 ± 21.70	
	Senior	44 (3.9%)	74.18 ± 22.16	
Any comorbid disease^b^				**0.015**
	**No**	**1,035 (92.7%)**	**71.94 ± 20.85**	
	**Yes**	**82 (7.3%)**	**79.59 ± 27.38**	

^a^ANOVA; ^b^independent sample *t*-test; Bold indicates a significant difference.

Table 2 reports on the means, standard, and correlations of the sample. Pearson’s correlation showed that cyberchondria has a significant positive correlation with stress and alexithymia (*r* = 0.50, *p* < 0.01, *r* = 0.36, *p* < 0.01). Further, it also demonstrates the significant positive correlation between alexithymia and stress (*t* = 0.53, *p* < 0.01).

**TABLE 2:** Means, standard deviations and correlations among alexithymia, stress, and cyberchondria (*N* = 1,117).

	Mean	SD	1	2	3	4	5	6	7	8	9	10
CSS	72.50	21.47	1									
CMP	15.10	6.91	0.93**	1								
DIS	17.23	6.35	0.93**	0.85**	1							
EXC	19.14	5.92	0.87**	0.71**	0.79**	1						
REA	13.51	4.78	0.83**	0.70**	0.72**	0.76**	1					
Stress	5.30	4.16	0.50**	0.50**	0.48**	0.40**	0.36**	1				
TAS	56.83	10.97	0.36**	0.33**	0.38**	0.35**	0.30**	0.53**	1			
DIF	17.81	5.17	0.45**	0.44**	0.45**	0.36**	0.32**	0.59**	0.86**	1		
DDF	14.10	3.10	0.29**	0.28**	0.31**	0.27**	0.25**	0.47**	0.88**	0.70**	1	
EOT	24.92	4.66	0.14**	0.10**	0.18**	0.24**	0.19**	0.29**	0.82**	0.45**	0.63**	1

CSS, Cyberchondria (CMP, compulsion; DIS, distress; EXC, excessiveness; REA, reassurance); TAS, alexithymia (DIF, difficulty identifying feelings; DDF, difficulty describing felling; EOT, externally oriented thinking); **Correlation is significant at the 0.01 level (2-tailed).

The result of linear regression analysis in Table 3 shows that stress (β = 0.50, *p* < 0.01) is an important positive predictor of cyberchondria. In addition, the analysis result shows that the overall model is significant (*F* = 377.79, *p* < 0.01), and 25% of the variation of cyberchondria is caused by stress.

**TABLE 3:** Regression analysis for predicting cyberchondria from stress (*N* = 1,117).

Predictor variables	*B*	*R* ^2^	*F*
Stress	0.50	0.25	377.79**

**p < 0.01.

Table 4 shows that alexithymia (β = 0.36, *p* < 0.01) is a positive predictor of cyberchondria, and in addition, the whole model is found to be significant (*F* = 160.46, *p* < 0.01), showing that 13% of the variation in cyberchondria is due to alexithymia. The linear regression results shows that DIF (β = 0.49, *p* < 0.01) could positively predict cyberchondria, EOT (β = −0.08, *p* < 0.01) could negatively predict cyberchondria, and DDF (β = 0.003, *p* > 0.05) could not predict cyberchondria. The results shows that 21% of the variation of cyberchondria is due to DIF and EOT (*F* = 97.77, *p* < 0.01).

**TABLE 4:** Regression analysis for alexithymia and its constructs as predictors of cyberchondria (*N* = 1,117).

	Predictor variables	*B*	*R* ^2^	*F*
(1)	TAS	0.36	0.13	160.46**
(2)	DIF	0.49**	0.21	97.77**
	DDF	0.003		
	EOT	−0.08*		

(1) = linear regression; (2) = multiple regression, **p < 0.01.

**FIGURE 1:**
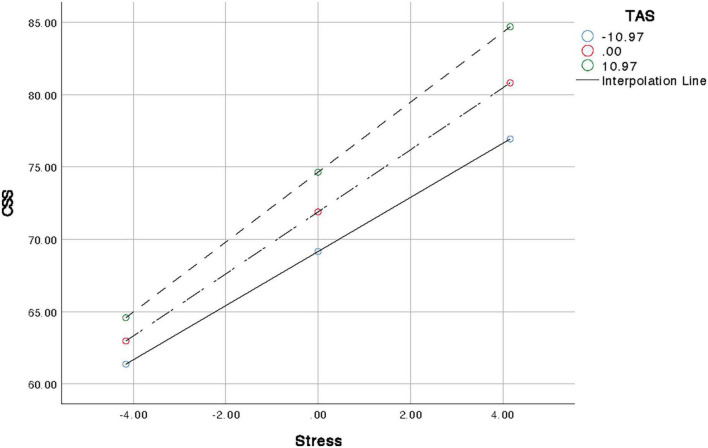
Graphical presentation of moderating role of alexithymia between stress and cyberchondria.

Table 5 shows the moderating effect of alexithymia between stress and cyberchondria. All continuous variables are standardized, and model analyses are performed after controlling for gender. The results shows that both alexithymia and stress have significant positive main effects on cyberchondria, and the interaction term have a significant predictive effect on cyberchondria. Alexithymia moderates the relationship between stress and network hypochondria (*F* = 107.20, *p* < 0.01), R2 = 0.28 shows that the moderating effect contribute 28% to the variation. Figure 1, further interaction analysis diagram, shows that with the increase of alexithymia level, the positive predictive effect of stress on cyberchondria becomes larger.

**TABLE 5:** Moderation of alexithymia between stress and cyberchondria.

Predictors	*B*	Cyberchondria	
		95%CI	
		LL	UL
(Constants)	78.12**	74.74	81.51
TAS	0.26**	0.14	0.37
Stress	2.12**	1.80	2.44
TAS × Stress	0.02**	0.01	0.04
*R* ^2^	0.28		
*F*	107.20**		

CI, confidence interval; LL, lower limit; UL, upper limit, **p < 0.01.

## Discussion

Based on the demographic results, it can be concluded that cyberchondria showed a greater symptoms expression in males and with physical or psychiatric illness. In previous studies, the results of cyberchondria were inconsistent in terms of gender factors during the pandemic. Some studies showed higher levels of cyberchondria in females (26, 27), while the Akhtar and Fatima’s (28) study was consistent with the present study. The investigation of psychological and mental health effects during COVID-19 found that women were more mentally burdened and had increased symptoms of depression and anxiety. However, how distress was relieved was also an important factor affecting cyberchondria. One possible reason for this result could be the age of the sample, which in this study was a college student group. Male college students had a higher prevalence of pathological internet use, which may result in excessive and repetitive health searches. Pathological internet use has important relationships with cyberchondria. Additionally, students with a physical or psychiatric disease were found to be associated with higher cyberchondria symptoms severity. The previous investigation studies have conducted that symptoms that have appeared recently and caused concern are triggers for online health searches. According to the most recent literature data (29), patients with psychiatric disorders (i.e., major depressive disorder, anxiety disorder, and OCD) have higher severity of cyberchondria symptoms. In this respect, the presence of a physical or psychiatric disease might be a predisposing factor for excessive searches for health-related information and for cyberchondria.

This study evaluated the relationship between stress, cyberchondria, and alexithymia. The results showed that stress was significantly positive correlated with cyberchondria and alexithymia (see Table 2). Based on the results of correlation analysis, regression analysis was carried out. The analysis showed that stress can significantly predicted cyberchondria, which supporting the first hypothesis. Individuals tend to seek information from different sources to confirm their health, when they are under stress, especially under health-related stress. During the COVID-19 pandemic, the internet has become one of the most common sources people use to search for health-related information, that 35% of individuals search for medical problems online in United States. Similarly, in a 2016 study, it was found that more than 50% of adults searched for health information online in the UK (30). However, such health-related online searches can sometimes increase individual anxiety and worry, making them trapped in repeated searches to ensure the accuracy of information, and eventually bringing up symptoms of cyberchondria (31).

Results of regression analysis show that alexithymia is a positive predictor of cyberchondria (see Table 4), supporting the second hypothesis. Among them, DDF (difficulty describing felling) can positively predict cyberchondria. Individuals with DDF are mainly characterized by the weakening of emotional awareness, which plays an important role in promoting the function of adaptive behavior (32). When individuals are exposed to negative emotional stimuli, it leads to the decoupling (mismatch) of subjective experience and physiological arousal. This decoupling may increase the risk of stress-related disorders in individuals with alexithymia, consistent with previous studies (33). In addition, alexithymia is an important risk factor for addictive behaviors, and studies have confirmed that alexithymia is associated with Internet addiction symptoms (34, 35). Previous findings showing that alexithymia increase the risk of PIU, and are a significant predictor of PIU. Individuals with higher alexithymia may be more likely to surf online when conducting health-related online searches, prioritizing online health research over other activities, leading to persistent or escalating negative consequences. The analysis results show that EOT is a predictor of CSS. EOT is mainly characterized by a tendency to focus on superficial information and avoid internal, affect-related thoughts.

The results also provide empirical support for the third hypothesis (see Table 5 and Figure 1) that alexithymia would enhance the relationship between stress and cyberchondria. The results show that the relationship between stress and cyberchondria is stronger when alexithymia is considered. Alexithymia plays an important role in the formation and maintenance of cyberchondria. When individuals are faced with stressors, especially health-related stressors, individuals with alexithymia tend to focus on and exaggerate their somatic feelings due to their weak emotional awareness (36), and then confirm their health status through health-related online searches. Additionally, individuals with alexithymia experience higher levels of emotional dysregulation, anger management and impulse control, and the problems increase the risk of excessive and maladaptive use of Internet. However, the information obtained by the search is mainly related to somatic sensations and physiological reactions, which is not completely applicable. After a brief reduction in the individual’s worry, the individual may experience increased distress and anxiety. To alleviate this anxiety, individuals search further online and become trapped in an endless cycle (3), which affects their daily lives.

This study has made some important contributions, but there were still some limitations we need to attach. Firstly, the data collection relies entirely on self-statement scales, so the final results may be influenced. Secondly, cross-sectional survey studies cannot draw casual inferences. Therefore, a longitudinal or experimental study should be conducted to obtain more convincing results. Third, the sample of this study was limited to the college student population, and the results may not be effectively generalized to other groups. Fourth, the presence of comorbidity in the demographic factors did not distinguish between organic and psychiatric disorders. In addition, apart from the moderating effect conducted by this study, more potential effects under this correlation can be further screened.

Despite the above limitations, this study also reaped many valuable fruits. This study has been designed to investigate the relationship between stress and cyberchondria beside the role of alexithymia. Analyses results show that stress is positively correlated with cyberchondria and alexithymia. In addition, stress and alexithymia are significant predictors of cyberchondria. Moreover, moderating analyses results show that alexithymia strengthen the relationship between stress and cyberchondria. This study is helpful for doctors and psychological workers to identify psychopathological factors and groups that are more vulnerable to cyberchondria when health-related stressful events and stressors appear. In addition, it is suggested that the development of adaptive emotion regulation strategies to regulate the influence of individual’s emotional recognition and attention to their own internal feelings when encountering stress can reduce the symptoms of cyberchondria, which provides a different perspective for the intervention of cyberchondria.

## Data availability statement

The raw data supporting the conclusions of this article will be made available by the authors, without undue reservation.

## Ethics statement

The studies involving human participants were reviewed and approved by the Institutional Review Board of the Third Xiangya Hospital Ethnic Committee (2017-S208). The patients/participants provided their written informed consent to participate in this study.

## Author contributions

YZ and LD designed the study, wrote the research protocol, supervised the survey, and checked the data. YZ, LD, and LY did the literature review, managed the field survey, quality control, and statistical analysis, prepared the manuscript draft. LD and YD contributed to the revisions in depth for the manuscript. All authors contributed to the article and approved the final manuscript.
